# Deciphering the Triple-Peak C-O-C Stretching FTIR Absorbance Consistently Occurring in Semicrystalline PEG

**DOI:** 10.3390/polym17162199

**Published:** 2025-08-12

**Authors:** Theodor Stern

**Affiliations:** Department of Chemical Engineering, Biotechnology and Materials, Faculty of Engineering, Ariel University, Ariel 40700, Israel; theodorst@ariel.ac.il

**Keywords:** PEG, FTIR spectroscopy, polyurethane, block copolymer, crystallization behavior, XRD, DSC, SEM

## Abstract

Polyethylene glycol (PEG) is among the most intensively researched and applied polymers, exhibiting a very wide range of industrial, pharmaceutical, and biomedical applications. The strongest and most highly diagnostic absorbance in the FTIR spectrum of PEG and of PEG-containing polyurethanes, is the ether C-O-C stretching absorbance, which consistently appears as a triple-peak absorbance in a semicrystalline state. Surprisingly, this phenomenon has very seldom been mentioned or elaborated, and no direct structural diagnostic FTIR assignment has been determined for each component of the triple-peak. The present research conclusively demonstrates that the left-side and right-side components of the triple-peak are assigned to the chain-fold regions and the extended-chain regions of the crystallized chains, respectively, while the strong-wide central component is assigned to the randomly oriented chains in the amorphous phase of the semicrystalline PEG. The present demonstration was facilitated via the synthesis of a highly oriented fibrillar polyurethane block-copolymer, exclusively containing extended-chain-crystallized PEG soft-segments, obtained through dense hard-segment crosslinking under vigorous unidirectional shear-stress continuously applied during the synthesis. The present research results enable us to directly relate the FTIR spectra of PEG and block copolymers synthesized thereof, to their crystallization mechanisms and chain conformations, thus facilitating the development of improved industrial processing methods.

## 1. Introduction

Polyethylene glycol (PEG) is among the most intensively researched and applied polymers, exhibiting a highly versatile and widespread range of industrial, pharmaceutical, and biomedical applications.

PEG is commonly hydroxy-terminated, with the chemical formula HO(CH_2_CH_2_O)_n_H and is commonly synthesized by ring-opening polymerization of ethylene oxide, using hydroxy anionic initiating catalysts, or via ring-opening polymerization of ethylene oxide with either H_2_O or ethylene glycol [1,2,3,4,5,6]. PEG can be synthesized in a very wide range of molecular weights, the most commonly researched and applied of which range between 200 and 35,000 g/mol [7,8,9,10].

Due to their low toxicity, biocompatibility, and inert nature, PEGs are frequently used in foods, cosmetics and pharmaceutical products [11,12,13,14,15] and particularly, PEGylated lipid nanoparticles are used in mRNA-based COVID-19 vaccines as delivery vehicles [7]. Chemical grafting of PEG to the surfaces of implantable biomedical devices and drug delivery systems has been widely researched and applied, since, when exposed to the aqueous biological fluids environment, the grafted PEG chains assume an extended brush-like configuration, in a direction normal to the grafting surface, thus inducing surface passivation and minimization of protein adsorption thereon [16,17,18,19,20].

PEG is also abundantly applied as soft segments in the synthesis of various types of block copolymers, especially in the synthesis of poly(ether urethane) elastomers [21,22]. These elastomers are typically synthesized via a two-step process, through the preliminary synthesis of a polyether-based (e.g., PEG) macro-diisocyanate prepolymer and subsequently chain-extended with a di-functional reagent being either a diol, a diamine, or a dicarboxylic acid resulting in block copolymers commonly termed poly(ether urethane urethane), poly(ether urethane urea), or poly(ether urethane amide), respectively [23,24,25,26], resulting in significantly elastic polymers, also exhibiting relatively high elongations at the break [27,28].

PEGs of low molecular weight, e.g., 200–600 g/mol, are transparent viscous amorphous liquids, in which the chains are in a quasi-random disordered orientation. PEGs of higher molecular weights, ranging from 1000 g/mol and above, are semicrystalline, consisting of both an amorphous phase and a crystalline phase in various ratios, depending mainly on their molecular weight and the thermal history [1,3,7,29]. All crystallizable polymers, including PEG, can never completely crystallize—and are thus always semicrystalline [30,31,32,33,34].

Crystallization of polymers, including PEG, typically forms platelet-like crystallites of nano-scale thickness—termed lamellae. There are only two possible crystallization mechanisms of polymer chains: 1. The extended chain mechanism—occurring via parallel unidirectionally stretched polymeric chains—a mechanism through which polymers mostly do not naturally crystallize, but it may be commonly induced by applying a strong tensile and/or shear stress on the polymer melt, before and during the crystallization process. This is highly applied in industry for the manufacturing of polymeric fibers by a spinning process [32,33,34,35,36]. 2. The chain folding mechanism—through which most polymers crystallize, by chain-folding in the direction normal to the lamellar plane [37,38,39,40,41].

The crystallization of PEG and the resulting structure, morphologies and properties have been extensively studied for many decades [2,41,42,43,44,45]. PEG was found to exhibit a monoclinic crystalline structure, while the chains within the crystalline regions were reported to assume a helical configuration, with two turns per repeating unit [3,42,43].

Although very distinct differences in the FTIR spectrum of PEG can be observed when measured in the semicrystalline state versus the amorphous state, this phenomenon has very seldom been mentioned or elaborated. A very well-known book entitled *Poly(ethylene oxide)*, published in 1976 [46], relates the observed differences in the FTIR spectrum of PEG in a semicrystalline state versus an amorphous state, to the presence of trans-gauche-trans helical chain rotation configurations, which are present in the crystalline state but not in the amorphous state [46]. Nevertheless, this has never been significantly further elaborated or demonstrated.

The strongest and most diagnostic absorbance in the FTIR spectrum of PEG is the ether C-O-C stretching absorbance. This absorbance is also the most prominent and highly diagnostic in the FTIR analysis of PEG soft segment-containing polyurethanes. The C-O-C stretching FTIR absorbance of semicrystalline PEG consistently exhibits a strong and sharp triple-peak absorbance, while only a strong single-peak C-O-C stretching absorbance is observed in amorphous PEG [46]. Since all three components of the triple-peak C-O-C stretching absorbance always appear in tandem in semicrystalline PEG, the correct assignment of each separate component of the triple-peak is not conclusively possible.

The present research focused on deciphering the source of each of the three components of the triple-peak C-O-C stretching FTIR absorbance in PEG, by experimentally creating the chemical and physical conditions under which the appearance of a peak component is inhibited in the semicrystalline state, thereby deducing the source of the remaining peak component in the spectrum.

It is hereby demonstrated that the left-side and right-side components of the triple-peak C-O-C stretching FTIR absorbance are assigned to the chain-fold regions and the extended-chain regions of the crystallized chains, respectively, while the strong central C-O-C stretching absorbance is assigned to the randomly oriented chains in the amorphous phase of the semicrystalline PEG. The demonstration and deciphering were facilitated by the synthesis and analysis of a polyurethane block copolymer, exclusively containing an extended-chain crystallized PEG soft-segment, obtained through dense hard-segment crosslinking under a vigorous unidirectional shear stress applied during the polymer synthesis.

The present research results enable the correct assignment and diagnostic interpretation of the FTIR spectrum of PEG and of block copolymers synthesized thereof—consequently enabling us to directly relate the FTIR spectra of these polymers to their crystallization mechanisms and chain conformations, thus facilitating the development of improved polymeric materials and industrial processing methods.

## 2. Materials and Methods

### 2.1. Materials

PEG200 (MW = 200, Sigma, St. Louis, MO, USA); PEG400 (MW = 400, Sigma); PEG6000 (MW = 6000, Sigma); Hexamethylene Diisocyanate (HDI) (Sigma); Glycerol (Sigma); Ethyl Stannous Hexanoate (Sigma); KBr powder for FTIR analysis (Sigma). (Sigma-Aldrich Israel Ltd., an affiliate of Merck KGaA, Darmstadt, Germany—Rehovot, Israel).

### 2.2. Instrumentation

FTIR analyses were carried out on a Perkin Elmer-Spectrum BX FTIR Spectrometer (Shelton, CT, USA). Measurements of PEG samples were performed as follows: Measurements of amorphous low-MW PEG (e.g., MW = 400) were performed by first preparing clean KBr pellets followed by spreading small amounts (8–10 mg) of the liquid PEG on the pellet surface, on an analytical scale. First, a background was measured, followed by the measurements of the PEG-surface-coated KBr pellets.

Measurements of the semicrystalline PEG samples (e.g., MW = 6000) were performed by first preparing clean KBr pellets followed by evenly spreading small amounts (8–10 mg) of the PEG powder on the pellet surface, on an analytical scale and horizontally placing it in the designated pellet holder. The PEG powder was then melted by placing said pellet in an oven, which was preheated to approximately 75 °C, until all the powder was completely liquified. The samples were then crystallized at room temperature on the KBr pellet. First, a background was measured, followed by the measurements of the PEG-surface-coated KBr pellets. Measurements of the synthesized polyurethane polymers were performed as follows: Small amounts of the polymer were first shredded using a metal file, after which the obtained polymer flakes were sieved using a fine sieve in order to obtain suitably small grains of the polymer. The polymer grains were then weighed (8–10 mg) and mixed with 150 mg of dried KBr powder. The mixture was then ground in a clean agate mortar, until a powdered mixture was obtained, from which KBr pellets were prepared. First, a background was measured, followed by the measurements of the KBr pellets. The FTIR instrument resolution was 4 cm^−1^. All spectra were measured in absorbance mode, in the range of 450–4000 cm^−1^.

Differential Scanning Calorimetry (DSC) thermal analyses were performed on a Mettler STARe SYSTEM DSC3 (Mettler-Toledo LLC, Greifensee, Switzerland), equipped with an FRS 5 sensor (Mettler-Toledo LLC, Greifensee, Switzerland), and an Intracooler Isolation Kit, with continuous nitrogen gas flow. Small amounts of the polymers or block copolymer were weighed (5–15 mg) in an aluminum crucible and sealed with a perforated lid. An identical sealed empty aluminum crucible was used as reference. The heating rate was 10 °C/min.

Polarized Optical Microscopy (POM) studies were performed on a Nikon H550S Optical microscope (Nikon, Tokyo, Japan), equipped with a C-SP Polarizer and D-DA Analyzer (Nikon, Tokyo, Japan), and an INVENIO 5SCIII 5M Pixel USB3 color digital camera (DeltaPix, Smorum Denmark), connected to Deltapix software (Deltapix Insight 2.5). Only the focus and light intensity were adjusted during the microscopy studies.

Scanning Electron Microscopy (SEM) studies were performed on a SEM JEOL 6510LV instrument (Tokyo, Japan), equipped with a SE (secondary electron) detector, with a resolution of 3nm at 30 KV. The acceleration voltage was 10KV. The polymers were viewed with Au sputter coating.

The X-Ray diffraction (XRD) pattern was obtained using XRD (SmartLab SE, Rigaku, Akishima, Japan) with Cu-K α radiation (λ = 1.5460 Å). Measurements were made in theta/2theta geometry (BBG—Bragg Brentano Geometry) in the range of 50–5 degrees at a step of 0.02 and a rate of 5 deg/min using a Kβ Ni filter. The phase/crystallinity analysis was performed using SmartLab Studio II ver. 4.2.44.0 with a Powder XRD/Compression module.

### 2.3. Polyurethane Syntheses

#### 2.3.1. Oriented Polyurethane Block Copolymer Synthesis

Polyurethane block copolymer, containing PEG200 segments, was synthesized in bulk, at a 1:1 PEG:HDI molar ratio.

Due to the fact that hexamethylene diisocyanate (HDI) may also react with water molecules, dry reaction conditions were provided via the following preparations: The PEG200 was dried at 105 °C under vacuum and magnetic stirring for 90 min, prior to synthesis. The glass reactor and stirring rod were also dried, in an oven at 105 °C for 90 min. The HDI was removed from refrigeration and kept at room temperature for 2 h prior to synthesis. The synthesis was performed under a continuous dry nitrogen gas flow, at a temperature of 80 °C.

A total of 50 g of polymer were synthesized, using the following steps: The dried PEG 200 was weighed directly into the glass reactor. The HDI was weighed separately and added to the reactor, under slow continuous mechanical stirring. Some 0.3 mL of catalyst (ethyl stannous hexanoate) was added to the reactor and the stirring speed was increased to the maximum speed that did not cause liquid splattering. The Teflon stirring-blade was fixed at very close proximity to the reactor glass wall—thus creating a very strong shear force in the reaction mixture and forming polymer, at the very high stirring speed applied. Thus, the polymer chains were consequently highly oriented in the stirring direction and immobilized in this state, following the simultaneous crosslinking process, due to the abundantly occurring side reactions. The synthesis was carried out until all the reactor content turned into a solid rubbery polymer. Additional unidirectional stretching of the still-hot polymer was triggered by the inevitable pulling of the polymer during removal from the reactor, leading to additional enhanced chain-extension and alignment. All analyses on diisocyanate-derived polymers should be performed after at least 3 months following synthesis completion, in order to ensure that the final properties of the polymer were obtained in terms of intermolecular and intersegmental bonds and crystallization.

#### 2.3.2. Highly Crosslinked Pure Polyurethane Synthesis

Synthesis of pure polyurethane was performed via the same procedure and conditions as described above for the block copolymer. The highly crosslinked pure polyurethane was synthesized with HDI and glycerol at a 1.5:1 molar ratio. A total weight of 50 g of polymer was synthesized. The synthesis is highly exothermal. Removal of the polymer from the reactor was performed while the polymer was still hot and the polymer became rock-hard after cooling.

The extremely high crosslinking density sterically inhibited the occurrence of all side-reactions; thus, a pure urethane-containing polymer was obtained.

## 3. Results and Discussion

All analyses of semicrystalline PEGs in the present research were performed on both PEG6000 and PEG10000. Since very similar results were obtained for both these materials, the results of PEG6000 are hereby representatively exhibited and described.

As also mentioned in the introduction section above, all PEGs exhibiting molecular weights ranging from and above 1000 g/mol are semicrystalline, i.e., containing both a crystalline phase and an amorphous phase.

**Figure 1:**
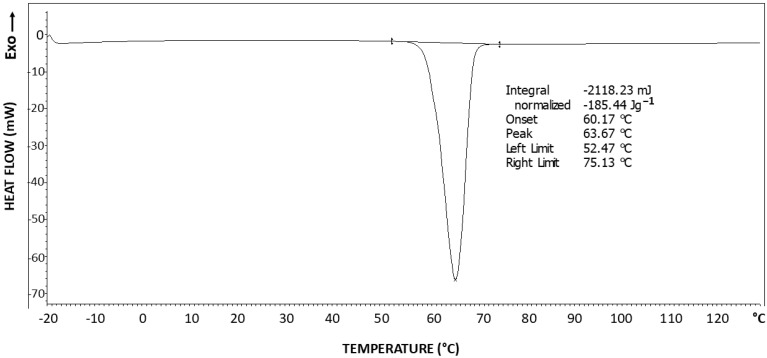
DSC thermogram of PEG6000.

Figure 1 exhibits the DSC thermogram of PEG 6000. The endotherm obtained at about 64 °C is characteristic of the melting of the crystalline phase of the PEG [46], with a melting enthalpy of ΔH = 185.44 J/g.

The theoretical melting enthalpy for 100% crystalline PEG is: ΔHm^0^ = 196.8 J/g [47]. Therefore, the degree of crystallinity of the PEG6000 is calculated according to Equation (1), by relating the hereby obtained melting enthalpy (ΔH = 185.44 J/g), to the ΔHm^0^ of PEG, resulting in a degree of crystallinity (Χ_c_) of 94.2% of the PEG6000. This relatively high degree of crystallinity is most probably due to the relatively low molecular weight of this polymer, leading to relatively low chain entanglements and relatively low melt viscosity, thus enabling us to obtain a relatively high degree of crystallinity. Nevertheless, the amorphous phase is still present in the polymer, as in all semicrystalline polymers.

Χ_c_ (%) = (ΔHm/ΔHm^0^) × 100 (1)

**Figure 2:**
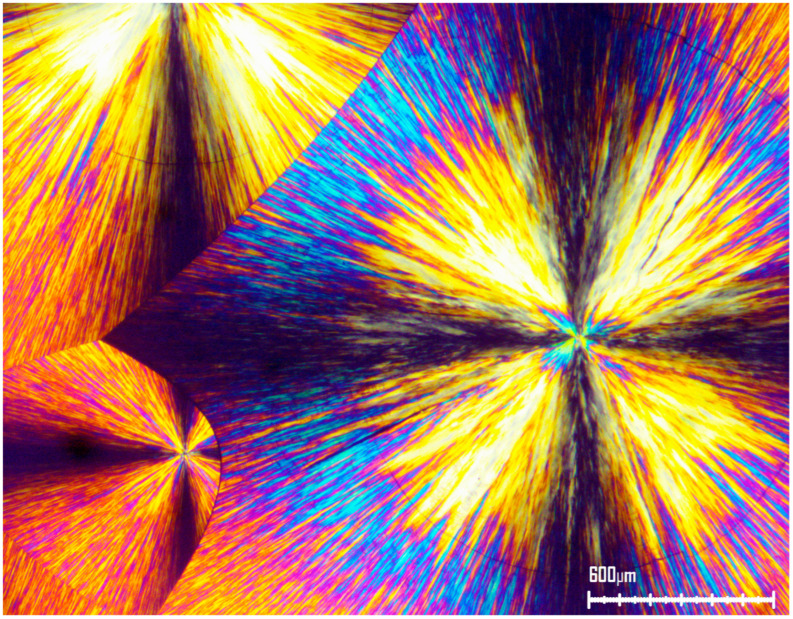
POM with crossed polars of PEG6000, exhibiting a quasi-radial symmetry of the lamellar structure of the spherulites (magnification 50×).

Figure 2 exhibits the POM image of PEG6000 crystals. The lamellar structure of the constituent crystallites can be clearly viewed. The black Maltese Cross extinction in each spherulite indicates the directions of the crossed polars.

**Figure 3:**
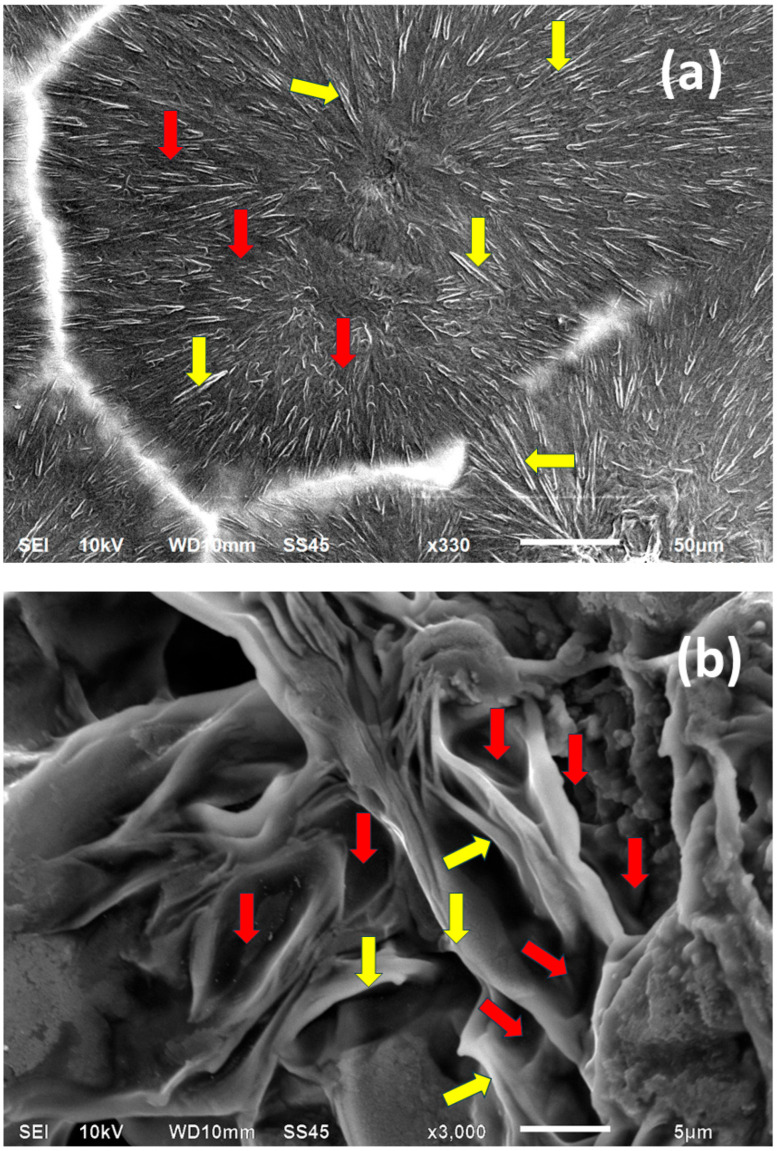
SEM micrograph of PEG6000 spherulites (magnification 330×) (**a**); and closeup high magnification of a representative branched lamellar structure occurring in the PEG6000 spherulites, enclosing interlamellar amorphous phase (magnification 3000×) (**b**). Representative crystalline lamellae are annotated with 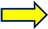; representative examples of enclosed inter-lamellar amorphous material are annotated with 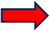.

Figure 3 exhibits the SEM scan of PEG 6000 spherulites. The quasi-radial symmetry of the lamellar structure in each spherulite is clearly visible. The crystalline lamellae appear light-to-white, as they much more strongly deflect the SEM electron beam towards the detector than the much softer amorphous phase, which appears very dark-gray or black in the SEM scan. Thus, it is clearly visible that the polymer (PEG 6000) contains both a crystalline phase and an amorphous phase, as expected in semicrystalline polymers. The amorphous phase accumulates at the lamellar surfaces and is enclosed in the interlamellar spaces (Figure 3).

**Figure 4:**
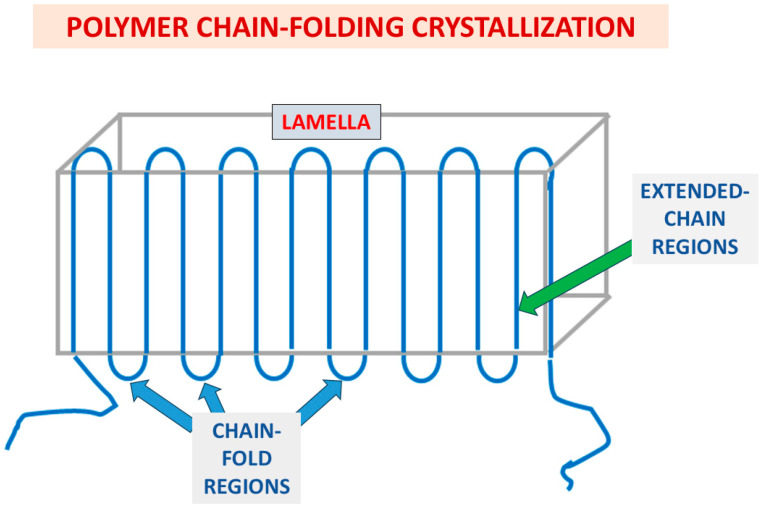
Schematic representation of the polymer chain folding crystallization mechanism, comprising both chain-folding regions and extended-chain regions. The amorphous phase occupies the inter-lamellar spaces (as viewed in the SEM scans presented above in Figure 3).

As described above in the introduction section, there are two possible crystallization mechanisms which can occur in crystallizable polymeric materials. The first is the extended chain mechanism—occurring via parallel unidirectionally stretched polymeric chains—and the second is the chain folding mechanism –in the direction normal to the lamellar plane. Figure 4 exhibits a schematic representation of the polymer chain folding crystallization mechanism, comprising both chain-folding regions and extended-chain regions. The amorphous phase occupies the inter-lamellar spaces, as viewed in the SEM scans presented above in Figure 3.

**Figure 5:**
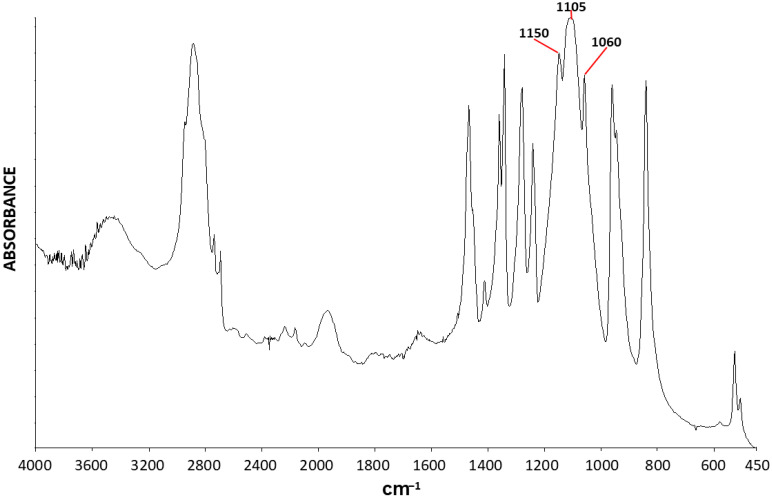
FTIR spectrum of PEG6000.

Figure 5 exhibits the FTIR spectrum of PEG6000. The C-O-C stretching vibration is the only strong absorbance of the ether group in the FTIR spectrum. Although two types of C-O-C stretching vibrations are known—i.e., the symmetric and asymmetric stretching vibrations—only the asymmetric stretching vibration can occur in symmetric ethers, including in PEG (which is a symmetric polyether), due to symmetry factors [48]. Thus, only a single strong ether C-O-C stretching absorbance peak is expected to occur in the FTIR spectrum of PEG. Nevertheless, in the FTIR spectrum of PEG6000 (Figure 5) a strong triple-peak C-O-C stretching absorbance is observed—consisting of three strong adjacent absorbances: at 1150 cm^−1^ (strong and sharp), at 1105 cm^−1^ (strong and wide), and at 1060 cm^−1^ (strong and sharp).

Additional absorbances in the spectrum belong mainly to vibrations of the PEG methylene groups: the symmetric and asymmetric C-H stretching at 2880–2950 cm^−1^; the asymmetric C-H bending at around 1460 cm^−1^ and the symmetric bending at around 1350 cm^−1^; and rocking vibrations at around 850 cm^−1^. The O-H stretching vibration of the hydroxy terminal groups occurs at around 3450 cm^−1^ and is weak due to the low concentration of these groups in the polymer.

Since, as discussed above, all crystallizable polymers are semicrystalline, including PEG—thus inherently also containing an amorphous phase—it may hereby be deduced that one component of the triple-peak C-O-C stretching absorbances should belong to the PEG chains in the amorphous phase of the polymer. In order to identify the C-O-C stretching absorbance peak assignment of the amorphous phase, the FTIR spectrum of a totally liquid amorphous low-molecular-weight PEG (i.e., PEG400) was measured.

**Figure 6:**
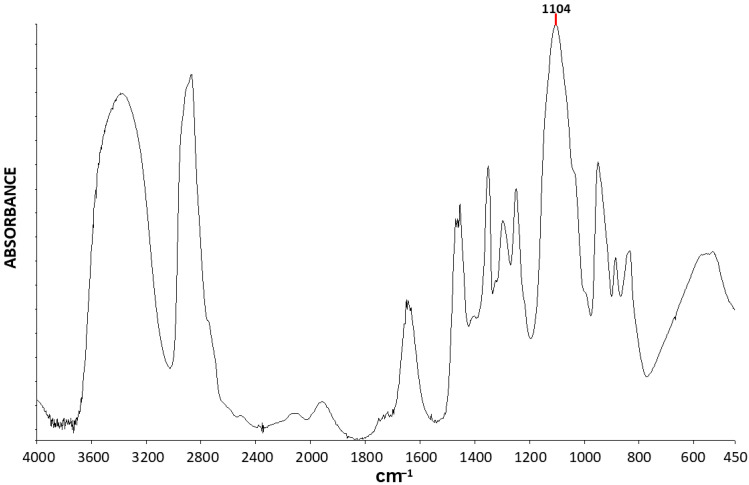
FTIR spectrum of PEG400.

Figure 6 exhibits the FTIR spectrum of PEG400. Only one clean single-peak strong ether C-O-C stretching absorbance is now observed in the spectrum, at around 1104 cm^−1^, which unambiguously indicates that in the spectrum of PEG6000 (Figure 5) the central peak component of the triple-peak, at 1105 cm^−1^, also belongs to the C-O-C stretching absorbance of the chains in the amorphous phase of the polymer. Additional peaks in the spectrum belong mainly to vibrations of the PEG methylene groups, as described above. The O-H stretching vibration of the hydroxy terminal groups occurs at around 3450 cm^−1^ and is now much stronger due to the much higher concentration of these groups in the lower molecular weight polymer.

In view of the above results, it is now clear that the two extreme-left and extreme-right strong and sharp absorbance components of the triple-peak of PEG6000 (Figure 5) belong to the C-O-C stretching absorbance of the chains in the crystalline phase of the polymer.

The question that inevitably arises here is to which region or configuration of the crystallized chains each of these two peak components can be assigned?

As the PEG6000 chains freely crystallized without any externally applied stress, it is evident that crystallization occurred via the chain-folding mechanism (and not via the extended-chain mechanism). Also, as described above, the polymer chain configuration in the chain-folded crystallized phase consists of two types of chain regions: 1. the chain-fold regions and, 2. the extended-chain regions (Figure 4).

It was hereby preliminarily hypothesized that each of these two peak components most probably belong to the C-O-C stretching vibrations occurring in either the chain-fold regions or the extended-chain regions of the crystallized chains of the PEG polymer.

The approach in the present research, to deciphering the correct and accurate diagnostic assignment of each of these two peak components, is through the synthesis and analysis of a polyurethane block copolymer, exclusively containing an extended-chain crystallized PEG soft-segment, obtained through dense hard-segment crosslinking under a vigorous unidirectional shear stress applied during the polymer synthesis.

The polymer was synthesized with hexamethylene diisocyanate (HDI) and PEG200, at an equimolar ratio, under a vigorous high-speed mechanical stirring, inducing strong parallel unidirectional chain-orientation and concomitant crosslinking thereof, through the inherent occurrence of diisocyanate-derived side reactions with already formed urethane groups.

As was previously conclusively demonstrated by the present author [49,50,51,52,53], the diisocyanate-derived polyurethane and polyurea synthesis is accompanied by predominant side-reactions occurring between isocyanate groups and the secondary nitrogens of urethane and urea groups, as the synthesis progresses, due to the inherently very high reactivity of the isocyanate groups [49,50,51,52,53]. It thus was further conclusively demonstrated that, once allophanate or biuret structures are formed, their newly formed N-H groups preferentially further consecutively side-react with additional isocyanate groups to form crosslinking tertiary oligo–uret structures, rather than forming additional new allophanate or biuret structures [49,50,51,52,53]. This occurs because the replacement of an H atom with a carbonyl in a urethane or in a urea group (thus forming an allophanate or a biuret, respectively) increases the electron-withdrawing activity towards the newly formed terminal N-H group, which in turn increases its polarity and thus also its reactivity towards an additional isocyanate group, leading to a preferential formation of the crosslinking tertiary oligo–uret network structures [49,50,51,52,53].

The highly oriented polyurethane block copolymer was recently synthesized by the present author [53], under the same conditions as described here, with the purpose of demonstrating the occurrence of side-reactions (in this polymer and in other polymers of the same family) and mainly, with the purpose of obtaining enhanced mechanical properties [53]. The remaining unused amount of the original 50 g of polymer synthesized was used as raw material and analyzed for the purpose of the present new research subject. Since the occurrence of the crosslinking side-reactions of exactly this polymer has already been thoroughly demonstrated [53] as well as of many other diisocyanate-derived polymers [49,50,51,52,53], this phenomenon will be only briefly discussed here. The sole purpose of the present research is specifically focused on deciphering the diagnostic assignment of the triple-peak components of the C-O-C stretching FTIR absorbance in semicrystalline PEG.

The synthesis of the polyurethane block copolymer, with hexamethylene diisocyanate (HDI) and PEG200, under a vigorous high-speed mechanical stirring, resulted in a strong flexible solid crosslinked highly oriented polymer.

**Figure 7:**
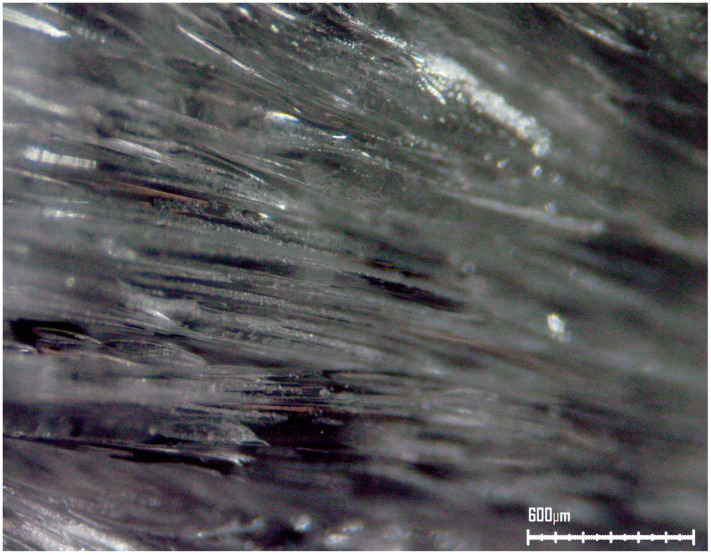
Optical microscopy photograph of a layer of the highly oriented synthesized fibrillar block copolymer. The very strong unidirectional orientation and the resulting abundant fibrous structures and fibrillation regions are clearly visible (magnification 50×).

Figure 7 exhibits an optical microscopy photograph of a layer of the block copolymer. The highly oriented structure of the polymer resulted in unidirectionally oriented fiber-like structures—even occasional fibrillation regions are observed, as are characteristically known to occur in polymeric fibers.

**Scheme 1:**
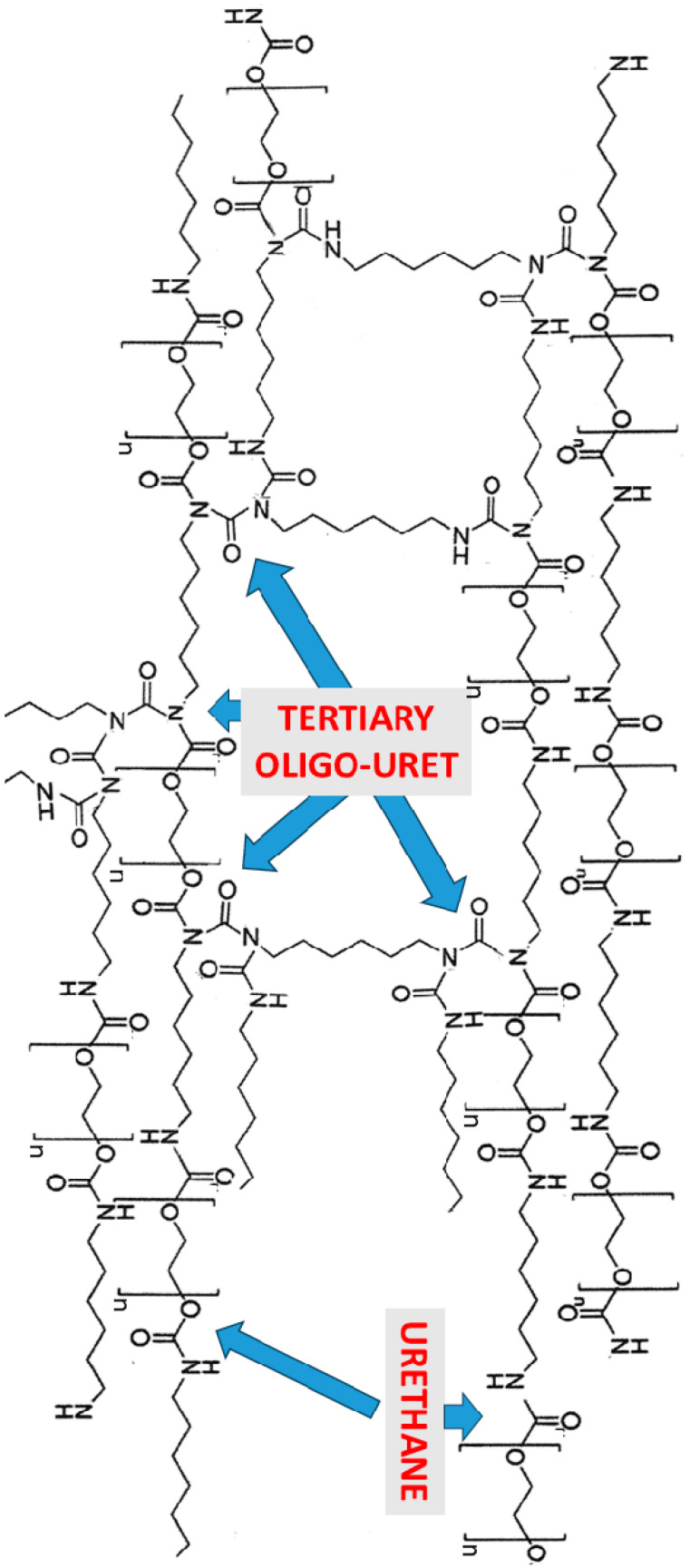
Schematic representation of the crosslinked hierarchical network formation, due to the concomitant preferential occurrence of side-reactions. Each of the NH groups in the structure may further react with an isocyanate, producing a tertiary nitrogen and a new secondary nitrogen which may react with an additional isocyanate (adapted from Stern T [53], MDPI, 2024).

Scheme 1 schematically exhibits the crosslinked hierarchical network formation in the polyurethane block copolymer synthesized with HDI and PEG200.

The occurrence of these crosslinking side-reactions was thoroughly and conclusively demonstrated in five recent pieces of research [49,50,51,52,53] for diisocyanate-derived polymers and for this type of polymer [49,50,51,52,53]. It was further identified and demonstrated [49,50,51,52,53] that the FTIR carbonyl-stretching absorbance is significantly different and consistently diagnostic for each of the diisocyanate-derived structure types, including the structures presented above in Scheme 1. The FTIR carbonyl-stretching absorbance of the pure urethane groups and pure urea groups and of the side-reaction products in diisocyanate-derived syntheses were determined via the development of new methods of total inhibition of the side-reactions and specifically of the tertiary oligo–uret structures, by steric hindrance [49,50,51,52,53], by the deliberate synthesis of a new polymer containing oligo–uret segments in the polymer repeating unit [52] and by the synthesis of diisocyanate-derived hyperbranched polymers with mono-amine-terminated and with mono-hydroxy-terminated reagents [51,53]. It was thus consistently demonstrated that the carbonyl-stretching FTIR absorbance of urethane groups occurs at 1717 cm^−1^ [49,50,53]; the absorbances of the two allophanate carbonyls occur at 1805 cm^−1^ and 1775 cm^−1^ [49]; the absorbance of the urea carbonyl groups at 1621 cm^−1^ [49,50,51,52,53]; of the biuret carbonyl groups at 1637 cm^−1^ [51,52]; and of the hierarchical tertiary oligo–uret network structure carbonyls at 1687 cm^−1^ [49,50,51,52,53].

This was in strong contrast with the many-decades-long paradigm described in some previous polyurethane-related research, which related the occurrence of two or more adjacent distinct carbonyl-stretching absorbance peaks in the FTIR spectrum to the combined presence and absence of hydrogen bonding—without even mentioning the highly probable occurrence of diisocyanate-derived side-reactions [54,55,56,57,58].

**Figure 8:**
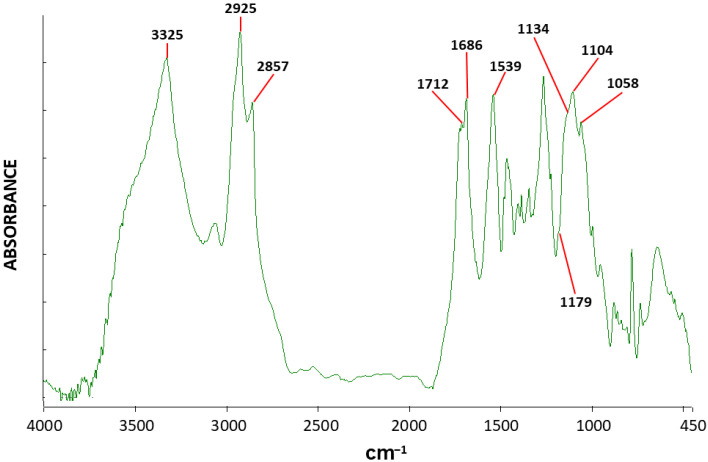
FTIR spectrum of the highly oriented fibrillar block copolymer synthesized. Only two C-O-C stretching peak-components appear in the spectrum.

Figure 8 exhibits the FTIR spectrum of the highly oriented polyurethane block copolymer. Two strong adjacent C=O stretching absorbances are clearly present in the spectrum: at 1711 cm^−1^ due to the urethane C=O stretching and at 1686 cm^−1^ due to the tertiary oligo–uret network C=O stretching (the tertiary oligo–uret network C=O stretching exhibits a slightly higher intensity than the urethane C=O stretching—thus, the tertiary oligo–uret network carbonyls exhibit a slightly higher abundance in the polymer than the urethane carbonyls). The slight shift in the urethane C=O stretching absorbance to a lower wavenumber, at 1711 cm^−1^ (as compared to a commonly occurring absorbance at 1717 cm^−1^), is most probably due to the more efficient hydrogen-bonding of these groups due to the close proximity of the parallelly oriented chains in this polymer. The very strong presence of the tertiary oligo–uret carbonyls in the spectrum clearly indicates the abundant formation of the crosslinking networks in this polymer. Additionally, the still significantly strong presence of the urethane groups’ carbonyl stretching absorbance in the spectrum (at 1711 cm^−1^) unambiguously indicates the strong preferential occurrence of the side reactions of the isocyanate groups with terminal NH groups in allophanate and tertiary oligo–uret structures, rather than reacting with additional available urethanes—which confirms the preferential side-reaction mechanism described above and in the recently published research [49,50,51,52,53]. The strong absorbance at 3324 cm^−1^ is due to the NH stretching of the non-further reacted urethane groups; the strong and sharp absorbance at 1538 cm^−1^ is due to the combined CNH deformation vibrations of the urethane groups; the strong absorbance at 1263 cm^−1^ belongs to the C-N-C stretching of the tertiary oligo–uret structures. The asymmetric and symmetric CH stretching absorbances are at 2925 cm^−1^ and at 2857 cm^−1^, respectively. The CH bending and CH rocking vibrations absorbances are at 1460 cm^−1^ and at 778 cm^−1^, respectively.

It is most important to note that the C-O-C stretching absorbance in the spectrum of this polymer exhibits only a double-peak (not a triple peak) consisting of only two components—at 1104 cm^−1^ (already attributed above to the disordered amorphous phase) and the right-side component of the peak at 1058 cm^−1^. The formerly observed left-side component of the triple-peak is now totally absent. The mere presence of the right-side peak-component at 1058 cm^−1^ is in itself a strong indication of the fact that the PEG segment of this polymer is semicrystalline (otherwise, only the central component at 1104 cm^−1^ would be present—as observed in the amorphous PEG400 in Figure 6 above). In addition, considering the strong chain orientation in this polymer, and in view of the fact that the PEG200 segments are too short for a chain-folding mechanism and, thus, can only crystallize in extended-chain conformation—this right-side peak component most probably belongs to the C-O-C stretching vibrations of the PEG chains crystallized via the extended-chain conformation. Thus, it may be deduced that the left-side peak component of the triple-peak most probably belongs to the C-O-C stretching vibrations of the PEG chains in the chain-fold conformation regions of the crystals in the higher molecular weight semicrystalline PEGs (e.g., PEG6000). The very slight shoulder at 1134 cm^−1^ is most probably due to the NC-O-C stretching absorbance of the urethane group in the block copolymer hard-segment. Another slight shoulder can be observed at around 1179 cm^−1^, and is most probably due to the ….N(C=O)N(C=O)N(C=O)NC-O-C urethyl NC-O-C stretching vibration strongly shifted to the left by the significant electron withdrawing effect of the adjacent tertiary oligo–uret structure. This will be further demonstrated below.

A solid-state ^13^C NMR spectrum of this same polymer can be observed in Stern T [53], exhibiting the occurrence of several different carbonyl types in the spectrum—which is highly consistent with the above-described tertiary oligo–uret crosslinking networks formation.

**Figure 9:**
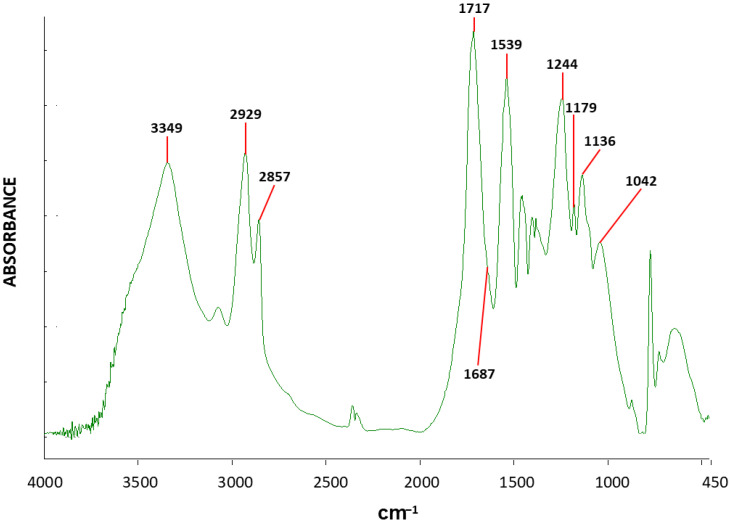
FTIR spectrum of the highly crosslinked polyurethane synthesized with HDI and glycerol.

Since the above-described polyurethane block copolymer consists of both PEG segments and urethane-containing segments—both exhibiting -O- stretching FTIR absorbances, the exact diagnostic location of the urethane-related -O- stretching absorbance needs to be determined and differentiated from the PEG-related C-O-C stretching absorbance. In order to further conclusively demonstrate the exact diagnostic location of the urethane-related -O- stretching absorbance, a pure polyurethane was synthesized through the complete sterical inhibition of all side reactions via an extremely high degree of crosslinking—by reacting HDI with glycerol at a molar ratio of 1.5:1—thus obtaining only pure urethane groups in the polymer (this polymer was first synthesized in Stern T [50]). Figure 9 exhibits the FTIR spectrum of the highly crosslinked polyurethane synthesized with HDI and glycerol. A single strong and clean carbonyl-stretching absorbance peak appears at 1717 cm^−1^, indicating that mainly urethane groups formed in the present polymer—and no side-reactions occurred. This is in strong contrast with the above-presented block-copolymer FTIR spectrum (in Figure 8), which additionally exhibits a strong C=O stretching absorbance at 1687 cm^−1^ due to the tertiary oligo–uret network structures-forming side-reactions. Nevertheless, a small shoulder at 1687 cm^−1^ can be observed in the spectrum, indicating that a small amount of tertiary oligo–uret structures did form in the polymer, most probably enabled by some regions of imperfect crosslinking.

The sharp urethane ether NC-O-C stretching absorbance peak of this almost pure polyurethane (Figure 9) appears at 1135 cm^−1^. This is in strong agreement with the above-observed slight shoulder at 1134 cm^−1^ attributed to the same urethane NC-O-C stretching absorbance (in Figure 8)—which occurs at a very significantly different absorbance than the strong-and-sharp left-side peak component of the triple-peak, at 1150 cm^−1^; the latter is totally absent in the FTIR spectrum of the above-described oriented block copolymer (Figure 8). In addition, a sharp absorbance peak can be observed at around 1179 cm^−1^, due to the assymetric NC-O-C stretching vibration of the tertiary oligo–uret urethyl group. This peak is in the exact same location as the slight shoulder viewed in the spectrum of the oriented block copolymer in Figure 8, which exhibits a very significant amount of tertiary oligo–uret crosslinking structures. A relatively small absorbance at 1042 cm^−1^ is due to the symmetric stretching absorbance of the urethane NC-O-C. All the above-described three components of the triple-peak are totally absent in the spectrum of the present highly crosslinked pure polyurethane (Figure 9), which is consistent with the fact that this polymer does not contain PEG.

Additional absorbances in the spectrum belong mainly to the urethane NH stretching absorbance at 3348 cm^−1^; vibrations of the HDI and glycerol methylene groups: the symmetric and asymmetric C-H stretching at 2857 cm^−1^ and 2925 cm^−1^, respectively; the C-H bending vibrations at around 1450 cm^−1^; the urethane HNC=O deformation at 1538 cm^−1^; and the C-N-C stretching vibrations at 1243 cm^−1^.

A solid-state ^13^C NMR spectrum of this same polymer can be observed in Stern T [52], exhibiting the occurrence of a single sharp carbonyl resonance peak denoting the occurrence of only one type of carbonyl in the spectrum—which is highly consistent with the above-described pure urethane formation in this polymer, due to the steric inhibition of the side-reactions.

**Figure 10:**
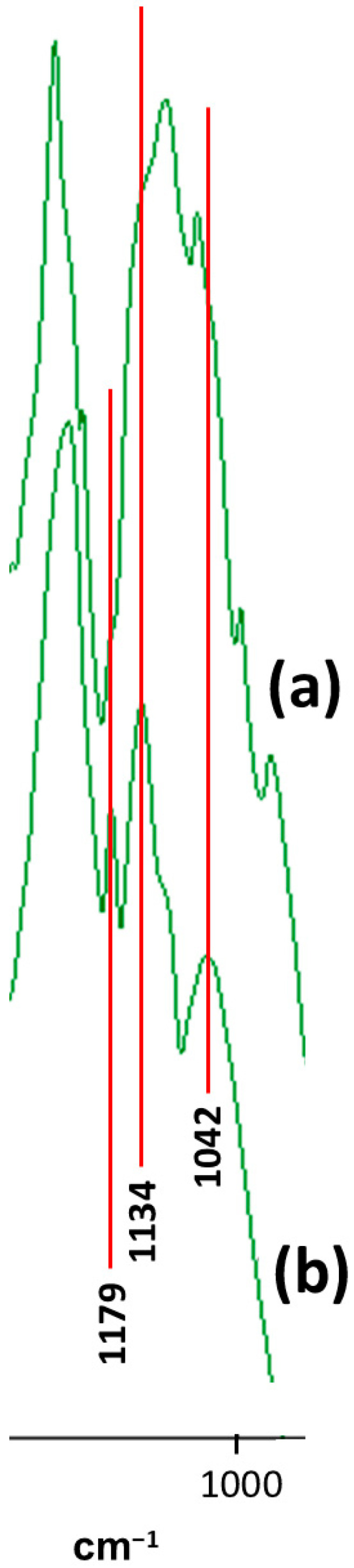
Expanded FTIR spectrum in the C-O-C stretching region of the highly oriented block-copolymer (**a**); and of the highly crosslinked polyurethane synthesized with HDI and glycerol, (**b**).

Figure 10 exhibits the expanded FTIR spectrum in the C-O-C stretching region of the highly oriented block-copolymer (a); and of the highly crosslinked polyurethane synthesized with HDI and glycerol (b). The vertical red lines clearly indicate that the three very small shoulders observed in spectrum (a), namely at 1179 cm^−1^, 1134 cm^−1^, and 1042 cm^−1^, coincide exactly in location with the absorbances of the tertiary oligo-uret urethyl NC-O-C stretching, asymmetric urethane NC-O-C stretching and symmetric urethane NC-O-C stretching, respectively—and are very significantly different in location to any of the PEG triple-peak components (of which the strong-sharp left-side component is totally absent in spectrum (a) of the block copolymer).

**Figure 11:**
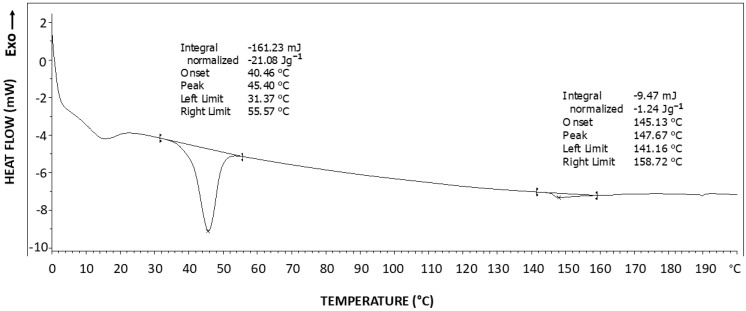
DSC thermogram of the highly oriented block copolymer synthesized.

Figure 11 exhibits the DSC thermogram of the highly oriented block copolymer synthesized. A significant melting endotherm is observed at about 45 °C, which is in the characteristic melting temperature range of the crystalline phase of PEG [46], with a melting enthalpy of ΔH = 21.08 J/g. For the theoretical 100% crystalline PEG, the melting enthalpy is: ΔHm^0^ = 196.8 J/g [47]. Accordingly, the PEG soft-segment degree of crystallinity may be calculated according to Equation (2), resulting in a PEG soft-segment degree of crystallinity of 19.65% (i.e., approximately 20% of the PEG200 soft segments have crystallized). Where MWPEG200 and MWRU are the molecular weights of the PEG200 soft segment and of the polymer stoichiometric repeating unit, respectively. In view of the fact that PEG200 in pure form is an amorphous liquid which cannot crystallize, this very significant soft segment crystallization in the present polymer occurred most probably due to the very close parallel proximity of some of the soft segments, fixed in a highly ordered extended-chain position by the crosslinking networks formed during the vigorous stirring shear stress applied during the synthesis process.

(2) $$Χc\;of\;PEG200\;segment\;(\%)=(\Delta Hm/{\Delta Hm}^{0})/(MWPEG200/MWRU)\times 100$$

An additional extremely small endotherm is observed at 147 °C, with an enthalpy of ΔH = 1.24 J/g, which is most probably the melting endotherm of a very small fraction of crystallized hard segments. In view of this extremely small melting endotherm obtained, the hard segments of this polymer may practically be considered amorphous—which may be explained by the fact that crosslinking occurs through the hard segments, most probably preventing the mobility required for their crystallization.

**Figure 12:**
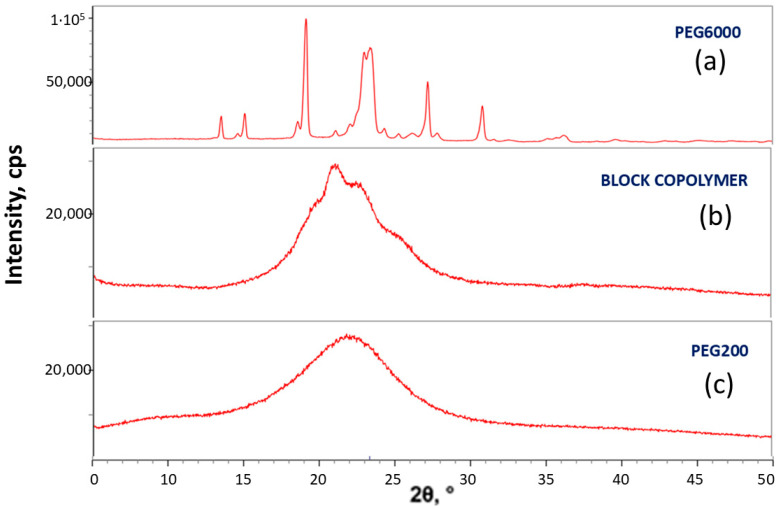
XRD pattern of PEG6000 (**a**); of the highly oriented polyurethane block copolymer synthesized (**b**); and of PEG200 (**c**).

Figure 12 exhibits the X-Ray diffraction (XRD) pattern of PEG6000 (a); of the highly oriented polyurethane block copolymer synthesized (b); and of PEG200 (c). The XRD pattern of PEG6000 (Figure 12a) is characteristic of the crystalline structure of PEG [46]. PEG200 is a totally amorphous liquid, which is reflected in its XRD pattern exhibiting only the broad amorphous peak (Figure 12c). The XRD pattern of the highly oriented polyurethane block copolymer synthesized (Figure 12b) clearly exhibits two crystalline peaks protruding above the broad amorphous peak, which correspond to the two highest-intensity peaks of PEG crystals. Although the degree of crystallinity is relatively low, it is clear from the XRD pattern that the PEG200 segments of the polymer have partially crystallized (i.e., are semicrystalline)—which is in strong agreement with the PEG melting endotherm viewed in the DSC thermogram of this block copolymer (Figure 11). Also, since the PEG200 segments are very short—consisting of only four ethylene oxide repeating units, which is not long enough for a chain-folding mechanism—the only possible crystallization mechanism in this case is the extended-chain mechanism, which is also in complete agreement with the highly unidirectionally oriented parallel fibrillar configuration of the chains in this block copolymer.

**Figure 13:**
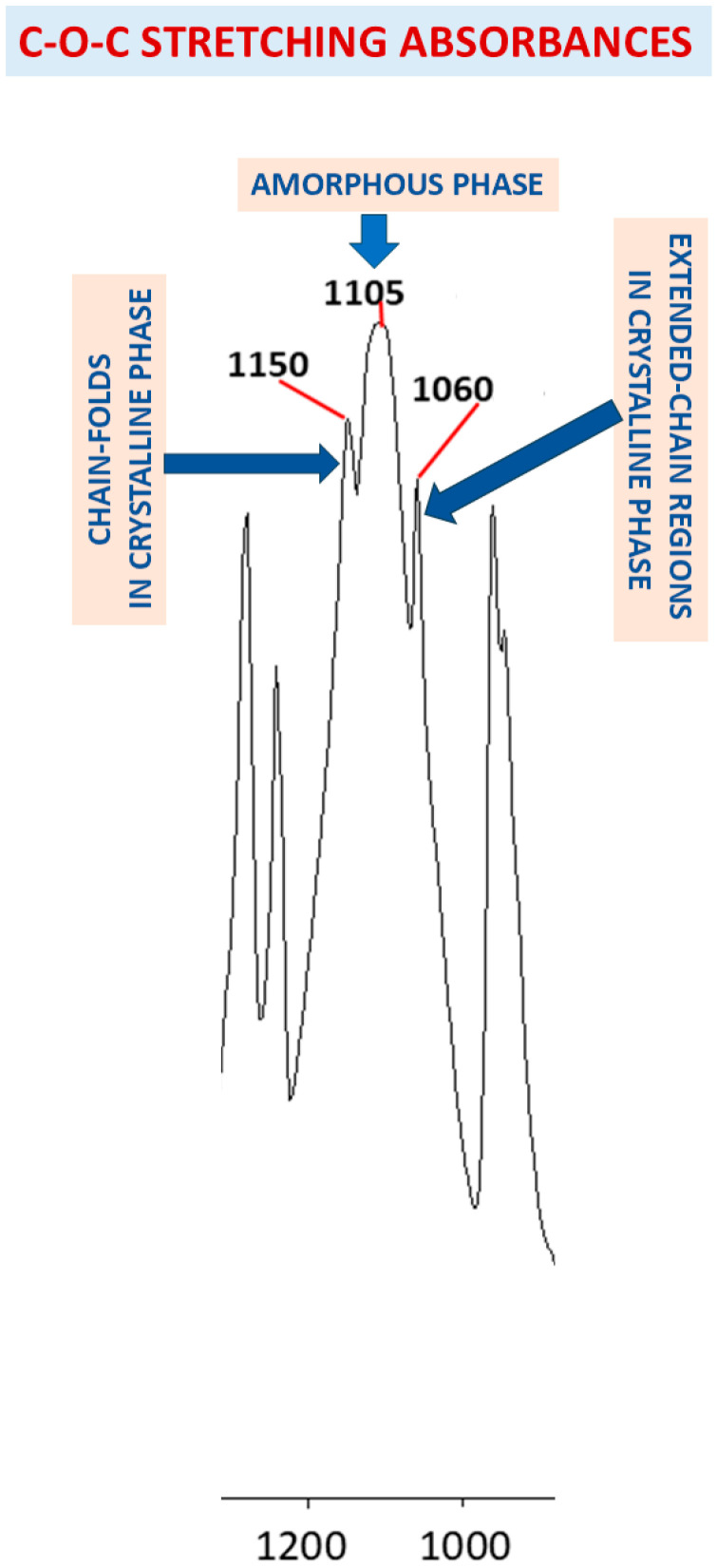
Diagnostic FTIR assignments of the triple-peak C-O-C stretching absorbance peak-components in PEG, as hereby demonstrated.

Figure 13 summarizes the demonstrated FTIR assignments, as related to the triple-peak C-O-C stretching absorbance peak-components in PEG. The left-side and right-side components of the triple-peak C-O-C stretching FTIR absorbance are assigned to the chain-fold regions and the extended-chain regions of the crystallized chains, respectively, while the strong-wide central C-O-C stretching absorbance is assigned to the randomly oriented chains in the amorphous phase of the semicrystalline PEG. This order of C-O-C stretching absorbance components of the triple-peak may also indicate that significantly more energy is required to produce a stretching vibration in a folded-chain region—resulting in an absorbance at a higher wavenumber, i.e., at a shorter wavelength, which relates to a higher absorbed energy. In an extended-chain region, significantly less energy is required to produce a C-O-C stretching vibration—resulting in an absorbance at a lower wavenumber, i.e., at a longer wavelength, which relates to a lower absorbed energy. The finding that the C-O-C stretching absorbance of the chains in the disordered amorphous phase occurs in the center between the left-side and right-side components of the triple-peak may stem from the possible wide range of configurations of the chains in the amorphous phase, which are neither extended nor folded; this also relates to the consistently wide shape of the central peak-component. Theoretical models which attempt at describing the effects of various parameters on peak intensities and location using charge redistribution and electro optical parameters such as atomic polar tensors are also known [59,60].

## 4. Conclusions

The semicrystalline state of PEG and its degree of crystallinity was hereby quantitatively first demonstrated by DSC thermal analysis. The combined presence of both a lamellar crystalline phase and an amorphous phase in the semicrystalline PEG was further confirmed and identified by SEM.

FTIR analysis of a totally liquid amorphous low molecular weight PEG enabled the identification of the C-O-C stretching absorbance of the chains in the amorphous phase, as the central component of the triple-peak—which appeared as a clean single-peak in the totally amorphous PEG.

Identification of the peak component related to the extended-chain regions of the crystallized chains of the PEG polymer was achieved through the synthesis and analysis of a highly oriented fibrillar polyurethane block copolymer, exclusively containing extended-chain crystallized PEG200 soft-segments, obtained through dense hard-segment crosslinking under a vigorous unidirectional shear stress applied during the polymer synthesis—thus, creating the chemical and physical conditions under which the appearance of the peak component belonging to the chain-folds is inhibited in the semicrystalline state—thereby conclusively also identifying the remaining peak component, as belonging to the extended-chain regions in the crystalline phase.

The exact diagnostic location of the NC-O-C stretching absorbance of the urethane group was hereby additionally demonstrated and determined by the synthesis and analysis of a highly crosslinked pure polyurethane, in which all side-reactions were sterically inhibited (and which did not contain PEG). Accordingly, it is hereby demonstrated that the NC-O-C stretching absorbance of the urethane group occurs at a totally different location than each of the C-O-C triple-peak components in PEG.

Optical microscopy revealed the highly oriented fibrillar structure of the polymer. Although PEG200 in its pure form can never crystallize, the semicrystalline state of the PEG200 soft segments in the synthesized highly oriented block copolymer was demonstrated and identified by DSC thermal analysis and XRD analysis.

It was hereby demonstrated that the left-side and right-side components of the triple-peak C-O-C stretching FTIR absorbance are assigned to the chain-fold regions and the extended-chain regions of the crystallized chains, respectively, while the strong-wide central C-O-C stretching absorbance is assigned to the randomly oriented chains in the amorphous phase of the semicrystalline PEG.

The present research results enable the correct peak-assignment and diagnostic interpretation of the FTIR spectrum of PEG and of block copolymers synthesized thereof—consequently enabling us to directly relate the FTIR spectra of these polymers to their crystallization mechanisms and chain configurations, thus facilitating the development of improved polymeric materials and industrial processing methods.

## Data Availability

The original contributions presented in this study are included in the article. Further inquiries can be directed to the corresponding author.

## References

[1] Thomas A., Müller S.S., Frey H. (2014). Beyond poly (ethylene glycol): Linear polyglycerol as a multifunctional polyether for biomedical and pharmaceutical applications. Biomacromolecules.

[2] Tadokoro H. (1967). Structure of crystalline polyethers. J. Polym. Sci. Macromol. Rev..

[3] Sánchez-Soto P., Ginés J., Arias M., Novák C., Ruiz-Conde A. (2002). Effect of molecular mass on the melting temperature, enthalpy and entropy of hydroxy-terminated PEO. J. Therm. Anal. Calorim..

[4] D’souza A.A., Shegokar R. (2016). Polyethylene glycol (PEG): A versatile polymer for pharmaceutical applications. Expert Opin. Drug Deliv..

[5] Harris J.M. (1985). Laboratory synthesis of polyethylene glycol derivatives. J. Macromol. Sci.-Rev. Macromol. Chem. Phys..

[6] Hu Y., Daoud W.A., Cheuk K.K.L., Lin C.S.K. (2016). Newly developed techniques on polycondensation, ring-opening polymerization and polymer modification: Focus on poly (lactic acid). Materials.

[7] Ibrahim M., Ramadan E., Elsadek N.E., Emam S.E., Shimizu T., Ando H., Ishima Y., Elgarhy O.H., Sarhan H.A., Hussein A.K. (2022). Polyethylene glycol (PEG): The nature, immunogenicity, and role in the hypersensitivity of PEGylated products. J. Control. Release.

[8] Daniela H., Frishberg M.D., Guo L., Darie C.C. (2014). Recent applications of polyethylene glycols (PEGs) and PEG derivatives. Mod. Chem. Appl..

[9] Zacchigna M., Cateni F., Drioli S., Bonora G.M. (2011). Multimeric, multifunctional derivatives of poly (ethylene glycol). Polymers.

[10] Ranjita S.H., Kamalinder S.I. (2010). In-vitro release of paracetamol from suppocire suppositories: Role of additives. Malays. J. Pharm. Sci..

[11] Strickley R.G. (2004). Solubilizing excipients in oral and injectable formulations. Pharm. Res..

[12] Harris J.M., Chess R.B. (2003). Effect of pegylation on pharmaceuticals. Nat. Rev. Drug Discov..

[13] DiPiro J.T., Michael K.A., Clark B.A., Dickson P., Vallner J.J., Bowden T.A., Tedesco F.J. (1986). Absorption of polyethylene glycol after administration of a PEG-electrolyte lavage solution. Clin. Pharm..

[14] da-Silva-Freitas D., Boldrini-França J., Candiani Arantes E. (2015). PEGylation: A successful approach to improve the biopharmaceutical potential of snake venom thrombin-like serine protease. Protein Pept. Lett..

[15] Ford J.L., Stewart A.F., Dubois J.L. (1986). The properties of solid dispersions of indomethacin or phenylbutazone in polyethylene glycol. Int. J. Pharm..

[16] Xiao X., Jiang X.Q., Zhou L. (2013). Surface modification of poly ethylene glycol to resist nonspecific adsorption of proteins. Chin. J. Anal. Chem..

[17] Stern T., Cohn D. (2001). Surface tailoring of biomedical polymers: An FTIR study. J. Appl. Polym. Sci..

[18] Cohn D., Stern T. (2000). Sequential surface derivatization of PET films. Macromolecules.

[19] Stern T., Penhasi A., Cohn D. (1995). Derivatization of a new poly (ether urethane amide) containing chemically active sites. Biomaterials.

[20] Wu J., Zhao C., Lin W., Hu R., Wang Q., Chen H., Li L., Chen S., Zheng J. (2014). Binding characteristics between polyethylene glycol (PEG) and proteins in aqueous solution. J. Mater. Chem. B.

[21] Yilgör I., Yilgör E., Wilkes G.L. (2015). Critical parameters in designing segmented polyurethanes and their effect on morphology and properties: A comprehensive review. Polymer.

[22] Sultan M., Masood R., Bibi I., Sajid I., Islam A., Atta S., Uroos M., Safa Y., Bhatti H.N. (2019). Impact of soft segment size on physicochemical and antimicrobial properties of waterborne polyurethane dispersions for textile applications. Prog. Org. Coat..

[23] Kircheldorf H.R. (1992). Handbook of Polymer Synthesis.

[24] De Souza F.M., Kahol P.K., Gupta R.K. (2021). Introduction to Polyurethane Chemistry, in: Polyurethane Chemistry: Renewable Polyols and Isocyanates. ACS Symp. Ser..

[25] Kanyanta V., Ivankovic A. (2010). Mechanical characterization of polyurethane elastomer for biomedical applications. J. Mech. Behav. Biomed. Mater..

[26] Mattia J., Painter P. (2007). A Comparison of Hydrogen Bonding and Order in a Polyurethane and Poly(urethane-urea) and Their Blends with Poly(ethylene glycol). Macromolecules.

[27] Tereshatov V., Vnutskikh Z., Slobodinyuk A., Makarova M., Senichev V. (2016). New multi-block isophorone diisocyanate-based copolymers with urethane urea hard segments. J. Elastomers Plast..

[28] Wong L., Dong X., Zhu P., Zhang X., Liu X., Wang D. (2017). High elasticity and corresponding microstructure origin of novel long chain poly(amide-block-ether) filament fibers. Eur. Polym. J..

[29] Zia F., Anjum M.N., Saif M.J., Jamil T., Malik K., Anjum S., BiBi I., Zia M.A. (2017). Alginate-poly (ethylene) glycol and poly (ethylene) oxide blend materials. Algae Based Polymers, Blends, and Composites.

[30] Bower D.I. (2008). Introduction to Polymer Physics.

[31] Sommer J.U., Reiter G. (2003). Polymer Crystallization—Observations, Concepts and Interpretations.

[32] Muhukumar M. (2007). Shifting Paradigms in Polymer Crystallization. Lect. Notes Phys..

[33] Piorkowska E., Rutledge C.G. (2013). Handbook of Polymer Crystallization.

[34] Zhang M.C., Guo B.-H., Xu J. (2017). A Review on Polymer Crystallization Theories. Crystals.

[35] Liu Q., Sun X., Li H., Yan S. (2013). Orientation-induced crystallization of isotactic polypropylene. Polymer.

[36] Hu W. (2023). Personal perspective on strain-induced polymer crystallization. J. Phys. Chem. B.

[37] Bassett D.C. (2003). Polymer Spherulites: A Modern Assessment. J. Macromol. Sci. Part B Phys..

[38] Woo E.M., Lugito G. (2015). Origins of periodic bands in polymer spherulites. Eur. Polym. J..

[39] Stern T., Teishev A., Marom G., Varelidis P.C., Papaspyrides C.D. (1996). Processing of Composites of Chopped PE Fiber-Reinforced PE Matrix. Adv. Compos. Lett..

[40] Stern T., Teishev A., Marom G. (1997). Composites of Polyethylene Reinforced with Chopped Polyethylene Fibers: Effect of Transcrystalline Interphase. Compos. Sci. Technol..

[41] Ren Y., Zou H., Wang S., Liu J., Gao D., Wu C., Zhang S. (2018). Effect of annealing on microstructure and tensile properties of polypropylene cast film. Colloid Polym. Sci..

[42] Allen R.C., Mandelkern L. (1982). Supermolecular structure of poly (ethylene oxide) fractions. J. Polym. Sci. Polym. Phys. Ed..

[43] French A.C., Thompson A.L., Davis B.G. (2009). High-Purity Discrete PEG-Oligomer Crystals Allow Structural Insight. Angew. Chem. Int. Ed..

[44] Stern T. (2017). Polymeric Micro-Sequential Concentric Transcrystalline Morphology Self-Assembly, with Intermittent Self-Shear-Oriented Amorphous Layers. Polym. Adv. Technol..

[45] Stern T. (2024). Transcrystalline Mechanism of Banded Spherulites Development in Melt-Crystallized Semicrystalline Polymers. Polymers.

[46] Bailey F.E., Koleske J.V. (1976). Poly(ethylene oxide).

[47] Bistac S., Brogly M., Bindel D. (2022). Crystallinity of Amphiphilic PE-b-PEG Copolymers. Polymers.

[48] Socrates G. (1994). Infrared Characteristic Group Frequencies: Tables and Charts.

[49] Stern T. (2018). Hierarchical fractal-structured allophanate-derived network formation in bulk polyurethane synthesis. Polym. Adv. Technol..

[50] Stern T. (2019). Conclusive chemical deciphering of the consistently-occurring double-peak carbonyl-stretching FTIR absorbance in polyurethanes. Polym. Adv. Technol..

[51] Stern T. (2020). Side-Reactions in Diisocyanate-Derived Bulk Polyurea Synthesis. J. Appl. Polym. Sci..

[52] Stern T. (2023). Chemical Structure and Side Reactions in Polyurea Synthesized via the Water-Diisocyanate Synthesis Pathway. Polymers.

[53] Stern T. (2024). Single-Step Synthesis and Characterization of Non-Linear Tough and Strong Segmented Polyurethane Elastomer Consisting of Very Short Hard and Soft Segments and Hierarchical Side-Reacted Networks and Single-Step Synthesis of Hierarchical Hyper-Branched Polyurethane. Molecules.

[54] Houton K.A., Wilson A.J. (2015). Hydrogen-bonded supramolecular polyurethanes. Polym. Int..

[55] Mc Kiernan R.L., Heinz A.M., Hsu S.L., Atkins E.D.T., Penell J., Gido S.P. (2002). Influence of hydrogen bonding on the crystallization behavior of semicrystalline polyurethanes. Macromolecules.

[56] Koberstein J.T., Gancarz I., Clarke T.C. (1986). The effects of morphological transitions on hydrogen bonding in polyurethanes: Preliminary results of simultaneous DSC-FTIR experiments. J. Polym. Sci. Part B Polym. Phys..

[57] Bistricic L., Baranovic G., Lescavac M., Bajsic E.G. (2010). Hydrogen bonding and mechanical properties of thin films of polyether-based polyurethane–silica nanocomposites. Eur. Polym. J..

[58] Ning L., De-Ning W., Sheng-Kang Y. (1997). Hydrogen bonding properties of segmented polyether poly (urethane urea) copolymer. Macromolecules.

[59] Galimberti D., Milani A., Castiglioni C. (2013). Infrared intensities and charge mobility in hydrogen bonded complexes. J. Chem. Phys..

[60] Dinur U. (1991). Charge flux and electrostatic forces in planar molecules. J. Phys. Chem..

